# Ordinary extraordinary: Elusive group differences in personality and psychological difficulties between STEM‐gifted adolescents and their peers

**DOI:** 10.1111/bjep.12349

**Published:** 2020-04-28

**Authors:** Maxim V. Likhanov, Elina S. Tsigeman, Kostas A. Papageorgiou, Aydar F. Akmalov, Ildar A. Sabitov, Yulia V. Kovas

**Affiliations:** ^1^ Sirius University of Science and Technology Sochi Russia; ^2^ Queen’s University Belfast UK; ^3^ International Centre for Research in Human Development Tomsk State University Russia; ^4^ Kazan Open University of Talents 2.0 Republic of Tatarstan, Russia; ^5^ Ulyanovsk State University Russia; ^6^ Department of Psychology Goldsmiths University of London UK

**Keywords:** academic achievement, behavioural strengths and difficulties, big‐ five, dark triad, giftedness, personality

## Abstract

**Background:**

Individual differences in personality, behavioural, and academic outcomes of gifted adolescents remain under‐explored.

**Aims:**

The present study directly compared selected and unselected adolescents on multiple measures of personality, behavioural strengths and difficulties, and achievement.

**Sample:**

Nine hundred seventy‐three adolescents selected for high performance in Science, Technology, Engineering, and Mathematical (STEM) fields (*M = *15.23; *SD = *1.11), and one thousand two hundred sixty‐one unselected adolescents (*M = *15.07; *SD = *1.18) participated in the study.

**Methods:**

Participants completed self‐report measures that assess the Big Five, the Dark Triad, and Behavioural Strengths and Difficulties. Demographic information and academic achievement in Maths and Russian were also obtained.

**Results:**

The observed differences in personality and behaviour traits between selected and unselected samples were negligible as measured by ANOVAs. The selected sample had on average slightly lower scores on conscientiousness, extraversion, agreeableness, openness to experience, and subclinical narcissism, partial Eta Squared (ES)* = *[.01 to .05]; slightly lower scores on prosocial behaviour; and slightly higher scores on internalizing and externalizing problems, ES* = *[.01 to .04]. The selected group also showed higher Year and Examination grades (ES* = *.05 and .23, respectively). However, MANOVA results showed larger differences between samples (ES* = *.15).

**Conclusion:**

Our results showed no pronounced differences between selected and unselected samples in any trait apart from examination performance. However, multivariate results suggest greater overall differences. These results suggest that high‐achieving individuals may be characterized by specific combinations of personality and behavioural traits.

## Background

Specialists in Science, Technology, Engineering, and Mathematics (STEM) contribute to national prosperity and the nation’s competitiveness in the international economy (Friedman, 2006; Friedman & Mandelbaum, 2012; Rinder & Thompson, 2011). Much effort is invested in development and implementing of talent identification methods and educational programmes aimed specifically at students showing high achievement in STEM subjects (Campbell & Walberg, 2011; Kell, Lubinski, & Benbow, 2013).

Selecting students for intensive educational programmes on school performance alone is as successful as the measures of achievement used. For example, in the Russian educational system, teacher ratings and most tests and examinations are assessed on a 2–5 scale, corresponding to fail–satisfactory–good–excellent ratings. Teacher assessments of students’ ability are likely to be affected by individual teacher’s standards for achievement; approaches, such as using grades for praise, punishment, and motivation; and conscious and unconscious biases, for example, reflecting children’s personality and behavioural characteristics (Campbell, 2015; Dompnier, Pansu, & Bressoux, 2006; Meissel *et al.*, 2017; Rausch *et al.*, 2016). Moreover, selection based only on achievement might overlook those, who potentially could have achieved more but had no opportunity yet, including those who cannot participate in domain‐specific competitions, for example, subject Olympiads (Liashenko, Khalezov & Arsalidou, 2017); and those overlooked by teachers due to ‘not fitting in socially’ (Geake & Gross, 2008).

Therefore, selecting on achievement may miss many talented youths, excluding them from programmes that can accelerate and promote their talent development. Much effort has been dedicated to creating standardized assessment instruments that evaluate cognitive ability and potential, such as verbal, numerical, and spatial (Kell, Lubinski, Benbow & Steiger, 2013; Shea, Lubinski & Benbow, 2001; Webb, Lubinski & Benbow, 2007). In addition, recent research has focused on non‐cognitive traits, such as personality, that also play a role in prediction of future academic and occupational achievement and well‐being (Farsides & Woodfield, 2007; Furnham, Monsen & Ahmetoglu, 2009; Heaven, Ciarrochi & Vialle, 2007; Lin, Clough, Welch & Papageorgiou, 2017; O’Connor & Paunonen, 2007).

Several studies have shown that there are differences in personality characteristics between unselected adolescents and those with high achievement (Limont, Dreszer‐Drogorób, Bedyńska, Śliwińska & Jastrzębska, 2014; Zeidner & Shani‐Zinovich, 2011). For example, Zeidner and Shani‐Zinovich (2011) found that high‐achieving students scored higher than unselected peers on openness to experience (Cohen’s *d = *0.51), but scored lower on neuroticism (Cohen’s *d = *−0.26) and agreeableness (Cohen’s *d = *−0.28). Several studies also explored the links between high cognitive ability and Dark Triad personality traits (Machiavellianism, subclinical psychopathy, subclinical narcissism; Paulhus & Williams, 2002). A recent meta‐analysis (O’Boyle, Forsyth, Banks & Story, 2013) investigated two hypotheses: That high‐performing individuals tend to display socially exploitative personality traits ‘evil genius’; or that lower‐performing individuals adopt manipulative behavioural tendencies to compensate for their disadvantages (‘compensatory’ hypothesis). These hypotheses were not supported by the meta‐analysis, with no robust links between Dark Triad (DT) traits and cognitive ability. However, other studies found some evidence for such links. For example, one recent study (Matta, Gritti & Lang, 2019) found some support for the ‘evil genius’ hypothesis as selected adolescents were more narcissistic, more callous (psychotic), and more suspicious (proxy to Machiavellian), with effect sizes of .10, .03, and .05, respectively. Another study (Papageorgiou, *et al*., 2018) also showed that DT traits might be linked to cognitive ability: The authors showed a longitudinal link between narcissism and school grades that was mediated by mental toughness.

High‐achieving adolescents may also differ from their peers in behavioural outcomes, such as inattention/hyperactivity or peer problems (Eren *et al.*, 2018; Neihart, 1999; Roedell, 1984; Terrassier, 2009). For example, their increased cognitive capacity may lead to problems in interpersonal relations and mental health (Neihart, 1999) via, for example, social withdrawal. However, a recent review has shown that high achievers did not demonstrate elevated levels of depression and suicide ideation, and even demonstrated lower anxiety than their peers (Cohen’s *d = *−0.72; Martin, Burns & Schonlau, 2009).

Establishing the differences in personality and behavioural difficulties between selected and unselected samples may be complicated by potential sex differences. For example, females demonstrate on average less conduct and peer problems, and report less hyperactivity and more prosocial behaviour, with Cohen’s *d* ranging from 0.13 to 0.68 (Eren *et al.*, 2018; Mieloo, *et al*., 2012). Average sex differences are also reported for academic achievement, with females on average outperforming males (Cohen’s *d = *[0.27–0.48]; Epstein, 1998; Gustavsen, 2019; Steinmayer & Spinath, 2008; Wong, Lam, & Ho, 2002; Van Houtte, 2004). A recent large‐scale study (*N* = 3.9 million; Reilly, Neumann & Andrews, 2018) showed that sex differences in academic achievement are mostly small‐to‐medium size, with other studies showing null differences (e.g., Voronin, Ovcharova, Bezrukova, & Kovas, 2018). Inconsistencies across studies may stem from differences in measures, as sex differences may be domain‐specific: Females on average show better results in verbal tasks; males show better results in some maths‐related tasks in both unselected (Stoet & Geary, 2013) and selected samples (Freeman & Garces‐Bascal, 2015). Such domain‐specific sex differences may emerge from average differences between males and females in specific cognitive abilities, as well as domain‐specific motivation to study, engagement in specific non‐academic activities and approaches to learning (Chamorro‐Premuzic & Furnham, 2008; Komarraju, Karau, & Schmeck, 2009; Willingham, Pollack, & Lewis, 2002). Sex differences in achievement may also be moderated by behavioural problems and personality. For example, in one study (Gibb, Fergusson & Horwood, 2008) sex differences in achievement were substantially reduced once teacher‐reported behavioural problems were controlled for (males were rated more inattentive, restless, and distractible than females).

In order to gain further insights into psychological processes underscoring high intellectual achievement, sex differences in personality, behaviour, and achievement need to be investigated in selected and unselected samples within a single study (see Freeman & Garces‐Bascal, 2015 for review). Only few such studies are available to date. For example, one study (Zeidner & Shani‐Zinovich, 2011) showed no significant interaction between sample (selected/unselected) and sex for personality traits. Females in both samples scored higher on neuroticism and agreeableness, while selected students showed more openness to experience and less neuroticism irrespective of sex. However, another study (Major, Johnson, & Deary, 2014) showed evidence for such interaction: The pattern of links between personality traits and general intelligence varied as a function of sex.

The current study explores differences between a large sample of adolescents selected on strict criteria for high achievement in STEM and their unselected peers on a wide range of measures. Given that this is the first study utilizing these two samples, we compared the two samples without formulating specific hypotheses. We explored:

1. differences between the two samples in personality (Big Five and Dark Triad); behavioural strengths (prosocial behaviour) and difficulties (externalizing and internalizing problems); and school achievement.
2. sex differences in these traits.

## Methods

### Sample

*Selected sample* included nine hundred seventy‐three students (577 males and 384 females; age: mean age* = *15.23, *SD = *1.11; range* = *14–18 years), recruited at the educational centre Sirius, in Sochi, Russia. Sirius provides intensive 24‐day educational programmes for high‐achieving adolescents (STEM, Art and Sport) from different regions of the country. An online application form is available for completion on Sirius website, inviting students to submit qualifying evidence. For the STEM track, selection criteria for the Centre admission are based on high performance in school subjects (biology, chemistry, mathematics, physics, etc.), as well as victory in subject Olympiads, good performance in university‐level ability/knowledge tests, developed specifically for each programme by organizers (top universities of Russia) and participation in school‐level conferences and scientific competitions (e.g., with a project). The selection is objective and does not rely on school, city, or region authorities’ recommendations. For each programme, all information is combined to create a ranking for each applicant, which is used to select best students for admission. For example, out of 22,000 applicants, 400 were selected for the STEM track in July 2019 (https://sochisirius.ru/news/2780). The ‘intellectually gifted’ status of these students is further supported by a recent finding of average advantage of a STEM‐selected sample at Sirius over university students from Russia and China in spatial ability tests (Cohen’s *d* equals 0.83 and 0.54, respectively; by comparing A. Budakovа, M. Likhanov, T. Toivainen, A. Zhurbitskiy, E. Sitnikova, E. Bezrukova, & Y. Kovas, unpublished data and Likhanov et al., 2018), as well as the same unselected school sample reported in another study (Cohen’s *d* ranging from 0.63 to 0.85; E. Tsigeman, M. Likhanov, A. Budakova, & Y. Kovas, unpublished data).

*Unselected sample* included one thousand two hundred sixty‐one students (626 males and 628 females; mean age* = *15.07, *SD = *1.18; range* = *14–18 years), recruited from several general education schools (no selection criteria) in different regions of the country via partner universities and schools.

### Procedure

Ethics Committee for Interdisciplinary Investigations, Tomsk State University (code of ethical approval: 16012018‐5) approved the study. Participants’ verbal consents and their parents’ or guardians’ written informed consents were obtained. Each participant completed online self‐report questionnaires in groups up to 25 people (an average number of students in school classes) under similar controlled conditions. The testing session lasted 1.5 hrs (two 45‐min school lessons in the Russian educational system). The participants did not receive feedback or compensation.

### Measures

Participants were asked to provide information on their age and sex, and (for the selected sample) on educational track (i.e., STEM) at Sirius. Next, participants completed three computerized instruments: The Big Five (John, Naumann & Soto, 2008), The Dark Triad (Jones & Paulhus, 2014) and The Strengths and Difficulties Questionnaire (Goodman, 2001; see Table 1 for detailed description of all measures); and provided information on three measures of their school achievement.

**Table 1:** Description of methods

Name	Description	Score computation	Russian adaptation and reliability
The Big Five (John, Naumann & Soto, 2008)	The Big Five Inventory (BFI) is a 44‐item questionnaire measuring the Big Five personality traits: openness to experience (e.g., ‘I see myself as someone who is original, comes up with new ideas’), conscientiousness (e.g., ‘I see myself as someone who is a reliable worker’), extraversion (e.g., ‘I see myself as someone who is talkative’), agreeableness (e.g., ‘I see myself as someone who is helpful and unselfish with others’), and neuroticism (e.g., ‘I see myself as someone who is depressed, blue’)	Participants reported their responses on a Likert scale ranging from 1 (strongly disagree) to 5 (strongly agree). Total scores for each trait were computed by averaging scores of corresponding items	This BFI version has been previously adapted to Russian by Shchebetenko and Wineshtein (2010) (including back translation and focus groups), was previously used with Russian samples (Shchebetenko, 2014), and showed high internal consistency for all scales (Cronbach’s α* = *.67 to .82; Mishkevich, 2016)
The Dark Triad (Jones & Paulhus, 2014)	The Short Dark Triad (SD3) is a 27‐item questionnaire assessing the Dark Triad of personality: narcissism, psychopathy, and Machiavellianism (Jones & Paulhus, 2014). Example items include ‘I like to use clever manipulation to get my way’ (Machiavellianism); ‘People see me as a natural leader’ (narcissism); and ‘It’s true that I can be mean to others’ (psychopathy)	Responses are given on a 5‐point Likert scale, with 1* = *strongly disagree and 5* = *strongly agree. The score for each subscale was computed by averaging corresponding items	The questionnaire has been adapted to Russian and demonstrated satisfactory reliability on a Russian adult sample, with Cronbach’s α > .70 for all three scales (Egorova, Sitnikova & Parshikova, 2015)
The Strengths and Difficulties Questionnaire (SDQ; Goodman, 2001)	SDQ is a behavioural screening questionnaire of adjustment and psychopathology for children and adolescents (4–17 years) (Goodman, 2001). The questionnaire has five scales that assess hyperactivity (e.g., ‘I am restless, overactive, cannot stay still for long’), emotional problems (e.g., ‘I worry a lot’), peer problems (e.g., ‘I am rather solitary, often play alone’), conduct problems (e.g., ‘I often have temper tantrums or hot tempers’), and prosocial behaviour (e.g., ‘I usually consider other people's feelings’)	Answers are provided on a 3‐point scale where 0* = *‘not true’, 1* = *‘somewhat true’, and 2* = *‘certainly true’. A score per scale is estimated by summing the responses to the five questions of each scale. A total behavioural difficulties score is a sum of all items, except the five items of the prosocial scale Given relatively low reliabilities for some scales and recommendations for analyses in low‐risk samples (Goodman, Lamping, & Ploubidis, 2010), a composite score of internalizing problems (emotional problems + peer problems) and a composite score of externalizing problems (hyperactivity + conduct problems) were used in the current study	The questionnaire was adapted to Russian language and showed satisfactory reliability for all scales (Cronbach’s α* = *.44 to .70; Ruchkin, Koposov, & Schwab‐Stone, 2007)

### School achievement

(1) *Year grade* was created as a composite, by averaging grades for two subjects: Russian language and Maths. The grades are teachers’ assessment of the students’ achievement during the academic year preceding data collection (reported by students). The grades range from one to five, but in practice ‘one’ is used very rarely and more as a punishment for student’s inappropriate conduct, rather than a measure of poor achievement. In the current sample, no one has received ‘one’. (2) *Examination* grade was created by averaging marks (range from one to five) for Year 9 State Examinations in two subjects: Russian language and Maths. This examination is taken at the end of 9th form (15–16 years of age) and is a standardized measure of students’ performance, serving as a major education assessment tool. Year 9 State Examination score ranges from 0 to 70 (the higher limit differs across academic subjects) and is then converted to scores from 2 to 5 to match the Year grades, using the state‐defined ranges (e.g., Examination score of 0–14, 15–24, 25–33, and 34–39 in the Russian language examination converts to 2, 3, 4, and 5, respectively). By the time of testing, only 447 and 363 participants for unselected and selected samples, respectively, have completed the 9th form (and taken the examination). Therefore, we did not include this variable in the multivariate analysis of variance (MANOVA), but ran a separate univariate analysis of variance (ANOVA). (3) *Olympiads* measure was based on participants’ report of the maximum level of Olympiads on the following scale: did not participate in the Olympiads (0), school (1), city/district (2), regional (3), federal (4), and international (5) Olympiads. To be admitted for an Olympiad of a greater level, an adolescent needs to win a top place at all previous levels. For example, to participate in a federal Olympiad one needs to win in school, city, and regional Olympiads.

### Statistical analysis

The data were pre‐processed and analysed using IBM SPSS (Statistical Package for the Social Sciences), version 23 software (IBM Corp., 2015). The univariate outliers were identified using z‐scores (a cut‐off of 3.29 was applied). Multivariate outliers (2 in unselected sample and 3 in selected sample) were identified using Mahalanobis distance and excluded from the analysis. After that, all study variables were approximately normally distributed within the samples, as per cut‐offs (absolute value of 2 for both skewness and kurtosis) suggested in George and Mallery (2010). Partial eta squared (Cohen, 1969) is used as an effect size measure (unless indicated otherwise) and is abbreviated as ES (effect size) throughout the paper.

## Results

### Descriptive statistics

Descriptive statistics for selected and unselected samples and reliabilities of study measures are presented in Table 2 and Tables S1 and S2.

**Table 2:** Descriptive statistics for personality, behavioural strengths and difficulties, and achievement

Sample	Unselected				Cronbach's α	Selected				Cronbach's α	Previously reported Russian samples*	
Sex (*N*)	Male (626)		Female (628)			Male (577)		Female (384)			Not divided by sex	
Statistics	Mean	*SD*	Mean	*SD*		Mean	*SD*	Mean	*SD*		Mean	*SD*
Conscientiousness	3.53	0.65	3.61	0.67	0.80	3.30	0.71	3.32	0.70	0.83	4.51	0.37
Extraversion	3.53	0.76	3.54	0.81	0.83	3.34	0.85	3.17	0.91	0.86	4.17	0.47
Agreeableness	3.63	0.54	3.76	0.57	0.70	3.47	0.59	3.59	0.62	0.74	3.93	0.52
Neuroticism	2.58	0.75	2.94	0.79	0.83	2.62	0.85	3.23	0.85	0.87	1.76	0.43
Openness to experience	3.59	0.61	3.78	0.66	0.78	3.51	0.65	3.71	0.66	0.79	4.29	0.45
Narcissism	2.96	0.54	2.96	0.54	0.70	2.86	0.57	2.79	0.59	0.74	2.78	0.73
Psychopathy	2.21	0.47	2.05	0.49	0.65	2.19	0.55	2.07	0.55	0.63	2.11	0.66
Machiavellianism	3.29	0.54	3.07	0.55	0.71	3.35	0.55	3.17	0.56	0.70	3.27	0.70
Prosocial scale	7.20	1.99	8.08	1.82	0.71	6.54	2.26	7.28	2.20	0.74	6.57	2.00
Internalizing problems	4.91	3.24	5.84	3.43	0.71	5.11	3.26	6.64	3.34	0.71	5.49	3.92
Externalizing problems	5.04	2.80	5.04	2.82	0.63	5.12	2.88	5.39	2.75	0.64	6.84	3.62
Behavioural strengths and difficulties	10.01	5.16	10.96	5.27	0.67	10.34	5.25	12.29	5.13	0.65	12.33	7.54
Year grade	4.23	0.65	4.46	0.64	–	4.63	0.38	4.80	0.35	–	–	–
Examination [*N*]	4.67 [226]	0.38	4.75 [221]	0.33	–	5.00 [186]	0.00	5.00 [170]	0.00	–	–	–

Notes

Sample sizes for all measures are presented in parentheses. The Examination sample sizes are specified separately in square brackets.

^*^ Means and SDs for Big Five scales are taken from Shchebetenko (2014); for Dark Triad from Egorova, Sitnikova and Parshikova (2015); and for behavioural strengths and difficulties from Ruchkin, Koposov, and Schwab‐Stone (2007). See Table 4 for descriptive statistics on Olympiads.

### Personality, behavioural strengths and difficulties, and academic achievement

To explore differences between samples and sexes in Big Five, Dark Triad, Strengths and Difficulties, and school achievement, a 2 × 2 MANOVA was conducted, with age as a control variable. Significant main effects were found for sample, Wilk’s λ* = *.85, *F* (12, 1729)* = *24.54, *p* < .001, ES* = *.15, and sex, Wilk’s λ* = *.75, *F* (24, 3458)* = *22.04, *p* < .001, ES* = *.13. An interaction effect between sample and sex factors was non‐significant.

Given that sex did not interact significantly with sample, we conducted 13 post‐hoc ANOVAs for the main effects of sample and sex alone for each of the study variables. Using the Bonferroni procedure, each ANOVA was tested at the significance level of .05/13* = *.004 level.

**Figure 1:**
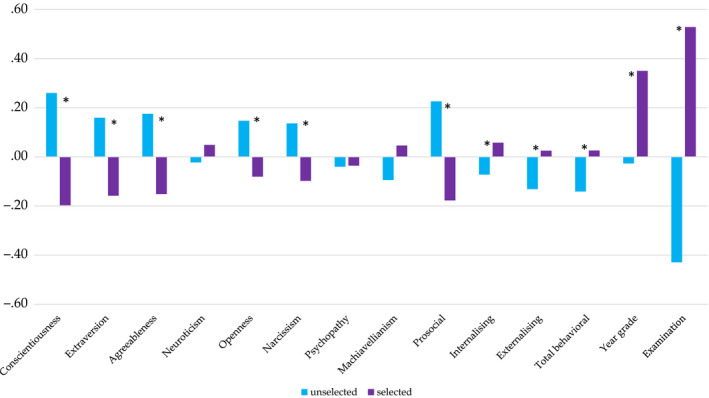
Mean differences between selected and unselected samples in the 14 study variables. Note: *p < .05; all means are given in standardized scores and allow for meaningful comparisons between variables; the prosocial scale of the SDQ is reversed, with greater scores corresponding to fewer problems.

The results are reported in Table 3 and Figure 1. Significant effects of sample were observed for 11 variables, with ES ranging from .006 to .055. On average, selected students showed lower scores on conscientiousness, extraversion, agreeableness, and openness to experience as well as lower scores on narcissism. Selected students also reported less prosocial behaviour, more internalizing and externalizing problems, more overall behavioural strengths and difficulties, and higher Year grades. An additional univariate ANOVA for Examination (on a reduced sample) showed that the selected sample had higher achievement in comparison with the unselected sample.

**Table 3:** Univariate ANOVA results for personality, behavioural strengths and difficulties, and achievement

	Sample			Sex			Sample*Sex	
	Difference score for sample	*F*	Partial η^2^	Difference score for sex	*F*	Partial η^2^	*F*	Partial η^2^
Conscientiousness	−0.32	89.70**	.049	−0.11	9.51**	.011	4.16	.002
Extraversion	−0.27	50.71**	.028	0.03	0.62	.001	3.42	.002
Agreeableness	−0.20	42.24**	.024	−0.15	13.28**	.015	0.03	.000
Neuroticism	0.06	7.53	.004	−0.49	82.96**	.087	8.07	.005
Openness to experience	−0.14	19.67**	.011	−0.22	23.49**	.026	.00	.000
Narcissism	−0.14	23.21**	.013	0.02	1.26	.001	0.89	.001
Psychopathy	0.00	0.27	.000	0.13	20.81**	.023	1.10	.001
Machiavellianism	0.08	7.04	.004	0.22	33.29**	.037	0.17	.000
Prosocial scale	−0.86	66.15**	.037	−0.91	36.83**	.041	1.36	.001
Internalizing scale	0.44	11.45*	.007	−1.27	35.36**	.039	2.07	.001
Externalizing scale	0.45	9.67*	.006	−0.09	8.01**	.009	3.30	.002
Behavioural strengths and difficulties	0.88	15.78**	.009	−1.36	22.74**	.025	3.87	.002
Year grade	0.21	101.56**	.055	−0.19	34.87**	.039	5.01	.003
Examination	0.29	235.62**	.227	−0.03	1.95	.005	4.08	.005

Notes

* *p* < .004 with multiple comparisons correction, difference score for sample was computed by subtracting mean for unselected sample from the mean for selected sample; difference score for sex was computed by subtracting mean for females from the mean for males.

** *p* < .001.

**Figure 2:**
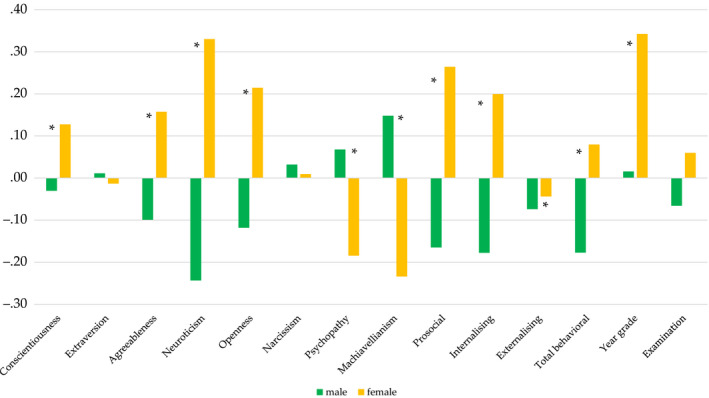
Mean differences between males and females in 14 study variables. Note: *p < .05; all means are given in Standardized scores; the prosocial scale of the SDQ is reversed, with greater scores corresponding to fewer problems.

The results for main effects of sex are presented in Table 3 and Figure 2. Significant effects were found for 11 of the variables, with overall negligible or weak effects, ranging from .01 to .09. The separate ANOVA for Examination (on a reduced sample) showed no sex differences.

### Year grade, examination performance, and Olympiad level

**Figure 3:**
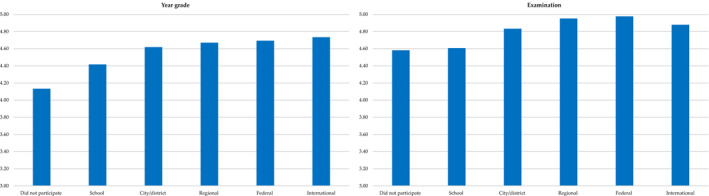
Mean Year and Examination grades of winners in different level Olympiads. *Note:* Selected and unselected samples were combined for this analysis. Panel to the left: Post‐hoc analysis with Tukey correction showed that those who did not participate in Olympiads received lower Year grades than winners at any other Olympiad level. School‐level winners had lower Year grades than those of city/district level or higher. Winners at city/district and higher levels did not differ from each other. Panel to the right: Post‐hoc analysis with Tukey correction showed that those who did not participate in Olympiads and winners at school level did not differ from each other, both groups received lower Examination grades than winners at city/district level or higher. Winners at city/district level received lower Examination grade than winners at regional and federal levels, but did not differ from winners at international level (*N = *14). Examination performance of those who won in regional, federal, or international levels did not differ.

The small differences (as per Cohen, 1969; Richardson, 2011) in Year grade between selected and unselected samples (ES* = *.06) may seem surprising, especially in the light of the relatively large differences between samples as per State Examination (ES* = *.23). We further explored the discriminative power of these measures by combining all participants into one sample and computing two univariate ANOVAs with victory in Olympiad being a predictor for Year and Examination grade (see Table 4 for number of Olympiad winners in relation to Examination and Year grade) (Figure 3).

**Table 4:** Number of Olympiad winners in relation to Year grade and Examination

Level of Olympiads	Unselected (*N* Year grade/*N* Examination)	Selected (*N* Year grade/*N* Examination)
Did not participate	122/53	7/2
School	267/134	14/6
City/district	305/152	208/40
Regional	150/67	383/173
Federal	51/26	300/128
International	34/15	61/14

Note

Participants were asked to acknowledge the maximum level of Olympiad they won in; selected and unselected samples differed significantly on the number of participants, who won in Olympiads of different levels (χ^2^ *= *634.14, *p* < .001).

We found that Year grade varied as a function of the Olympiad level, Welch (5, 490.98)* = *24.09, *p* < .001, ES* = *.09. Welch test was used, as the assumption of variance homogeneity across groups was violated, Levene’s statistic (5, 1864)* = *18.36, *p* < .001. For Examination grade, the effect was stronger: Welch (5, 174.05)* = *37.38, p <.001, ES* = *.24. Variance homogeneity assumption was also violated for this analysis, Levene’s statistic (5, 804)* = *92.67, *p* < .001. See Figure 3 for post‐hoc comparisons and means.

**Figure 4:**
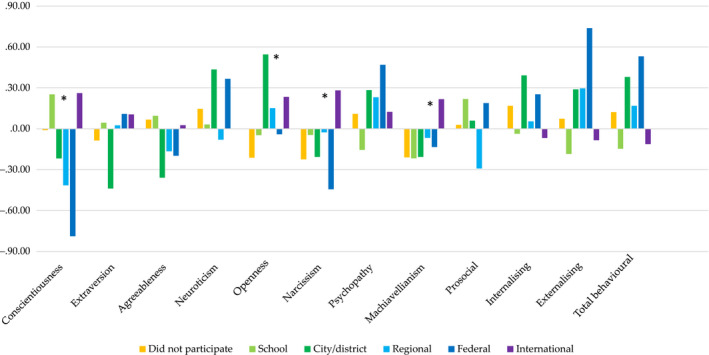
Mean differences between winners of different level Olympiads in the 12 study variables. Note: *p < .004; all means are given in standardized scores (*z*‐scores).

In addition, we conducted a separate MANOVA to explore whether Olympiad winners at different levels differed in personality and behavioural strengths and difficulties that showed negligible differences (ES did not exceed .01; see Figure 4). More details on the analysis are available from authors.

## Discussion

The current study investigated differences between a large sample of adolescents selected on strict criteria for high achievement in STEM and their unselected peers in personality, behavioural strengths and difficulties, and academic achievement. Consistent with previous research (e.g., Zeidner & Shani‐Zinovich, 2011), we found no interaction between selection and sex. This suggests that the observed differences between the selected and unselected samples did not result from differences in sex composition of the samples; and that the observed differences between males and females did not result from differences between the samples. The results showed significant, but weak main effects of both sample (selected/unselected) and sex (male/female) on most measures.

### Differences between selected and unselected samples

Multivariate analysis showed relatively large difference between selected and unselected samples (ES* = *.15). However, when personality measures were examined individually, differences between the samples in personality were small (.01 to .05), suggesting that individual personality traits are not robust independent predictors of high academic achievement. The selected sample scored lower than the unselected sample on extraversion, conscientiousness, agreeableness, and openness to experience (no differences in neuroticism). The slightly lower scores of the selected sample on extraversion are in line with previous research, e.g. Pincombe, Luciano, Martin, and Wright (2007). This may reflect the tendency of gifted students to study rather than to participate in social activities; or their tendency for selecting study over socializing – contributing to development of intellectual giftedness (e.g., Major *et al.*, 2014). Higher conscientiousness in the unselected sample is also in line with previous findings (Chamorro‐Premuzic & Furnham, 2006; Moutafi*et al.*, 2003), and may reflect using conscientiousness to compensate for lower intelligence.

Previous research on the links between conscientiousness and intelligence is inconsistent, showing no differences, higher and lower conscientiousness in selected samples than unselected samples (see Zeidner and Shani‐Zinovich (2011) and Major *et al.* (2014) for discussion). Moreover, the overall means of personality traits differ across studies. A possible explanation for lower conscientiousness in the selected sample in our study is differences in the frame of reference. The frame of reference for unselected adolescents is normal distribution of conscientiousness that they see in their peers. In contrast, the selected schoolchildren in Sirius may be influenced by their exposure to a group of highly selected adolescents, which may lead to lower self‐perceived conscientiousness (i.e., being a small fish in a big pond; Fang *et al*., (2018)). In addition, the lower conscientiousness in gifted adolescents may result from their negative experiences with peers. For example, 67% of gifted learners surveyed reported that they had experienced bullying (Peterson & Ray, 2006), which may cause them to hide their positive personality traits. Furthermore, inconsistences in results across studies may also be related to differences in cultural norms in regard to making positive statements about one’s skills and abilities and expressing positive emotions (Sheldon, *et al.*, 2017). However, this explanation is inconsistent with at least one other study with an unselected Russian sample (Shchebetenko, 2014) that found higher means in, for example, conscientiousness and agreeableness than in our study. As our sample was younger, further research is needed to disentangle cultural and other sampling explanations.

Our results on agreeableness are in line with some previous research (e.g., Zeidner and Shani‐Zinovich (2011)) that found lower agreeableness in selected adolescents. We found negligible differences in openness to experience and no differences in neuroticism. This contrasts with previous research that found less neuroticism and more openness to experience in the selected samples (McCrae, *et al.*, 2002; Zeidner & Shani‐Zinovich, 2011). However, the effect was negligible (0.01) and requires further replication. As with conscientiousness, further research is needed to understand the sources of these inconsistencies.

In line with results of the meta‐analysis (O’Boyle *et al.*, 2013), our data showed no robust link between Dark Triad personality traits and giftedness. The selected sample was lower in narcissism (ES* = *.01), but did not differ from unselected students in Machiavellianism and psychopathy. The observed small difference in narcissism but not the other two traits supports the view of its distinct place in the Dark Triad, and in fact its positive and protective role in academic achievement and mental health under certain circumstances (see Papageorgiou, Denovan, & Dagnall, 2019; Papageorgiou, *et al*., 2019; Papageorgiou, *et al*, 2018; Papageorgiou, Wong, & Clough, 2017).

The selected sample demonstrated slightly more behavioural difficulties and less prosocial behaviour, ES* = *[.01 to .04]. These small differences may stem from multiple processes. For example, selected students have a high rate of participation in ‘high‐stakes’ educational programmes and academic competitions (Embse & Hasson, 2012), as well as high expectations from parents/teachers – which may lead to greater levels of anxiety (Margot & Rinn, 2016). However, further speculations on the observed differences are premature as the body of existing research on this is inconsistent (for review, see Yildiz, Altay, & Toruner, 2017). For example, one study (Guénolé, *et al*., 2013) also showed that gifted children demonstrated more externalizing and internalizing problems (Cohen’s d equals .88 and .84, respectively). Another study reported that gifted students had more internalizing problems (Cohen’s *d* equals .71) but did not differ from non‐gifted sample in externalizing problems (Morawska & Sanders, 2008). Yet, another study showed no differences in behavioural problems between gifted and non‐gifted samples, except for greater hyperactivity/inattention in gifted students (Cohen’s *d* equals 0.42; Eren *et al.*, 2018). Overall, the existing evidence, including the current results, does not support the widely held view of excessive behavioural problems in gifted adolescents.

In regard to academic achievement, we found higher Year grade and Year 9 State Examination in selected sample. This is consistent with previous data, showing greater average performance on the Year 11 State Examination of adolescents attending specialist maths school than of those attending regular schools (Voronin *et al.*, 2018). However, the effect sizes in Voronin and colleagues (Cohen’s *d = *3.26 and 1.48 for Math Examination and Russian Examination, respectively) are greater than those in the present study (partial eta squared converted to Cohen’s *d = *0.48 for Year grade and 1.08 for Year 9 State Examination). The smaller effect in our study is likely a result of differences in the grading of the two examinations: Year 9 State Examination grades are converted to scores from 2 to 5 which makes this measure less sensitive (e.g., 95% of the selected sample had the top grade ‘5’). In contrast, Year 11 Examination is marked on the 0‐100 scale and is not converted – allowing for greater differentiation at the top end of ability.

Our study also showed that the observed sample differences were greater for the Year 9 State Examination than for the Year grade (.23 vs. .06, respectively). A possible explanation for these differences is that Year grade may not fully reflect objective achievement as teachers can build rewards for effort and punishment for lack of effort or motivation into the grade, as well as personality characteristics of students, such as conscientiousness. Consequently, a student with weaker knowledge, but high motivation and effort may be rewarded with a higher grade than a student with stronger knowledge but low motivation. In addition, in the present study the selected sample was on average less conscientious than the unselected sample, which could have led to a lower overall teacher rating for the selected sample. This is further supported by the presence of a positive correlation between Year grade and conscientiousness in selected (.21) and unselected (.20) samples. However, an inability of Year grade to distinguish between selected and unselected samples indicates its limited ability to assess real achievement. In contrast, the state examination is not subject to this limitation, as these examinations are usually sat at a different location from the students’ schools and the papers are evaluated anonymously by other teachers.

This interpretation is supported by another study from the same population (including adolescents with high achievement in Science, Sport, and Art; Papageorgiou, *et al*., 2020), where neither Dark Triad, nor Big Five personality traits predicted the Year 9 State Examination, while explaining some variance in the Year grade. This is inconsistent with previous studies. For example, in a study of UK unselected adolescents, a correlation of .24 was found between the standardized examination and conscientiousness (Rimfeld, Kovas, Dale & Plomin, 2016). Moreover, a relatively low correlation between Year 9 State Examination and Year grade (*r = *.55; Papageorgiou, *et al*., 2020) is also inconsistent with research in other countries and might be another point of concern. For example, in a UK sample a stronger correlation (*r = *.85) was found between a composite standardized Examination grade at age 16 and composite teacher rated Year grade at age 14 (Rimfeld *et al.*, 2019). The Russian grading system might be too crude to detect real correlations with personality (e.g., a causal link between conscientiousness and achievement) and instead may reflect some subjectivity of teacher ratings. However, these explanations are speculative and more research is needed to directly test these hypotheses, especially since sample sizes differed for Examination and Year grade in the current study.

We also examined the data on subject Olympiads and found that while there were proportionately more school (21.3 vs. 1.4), city/district‐level (24.3 vs. 21.6) Olympiad winners, and those who did not participate in any (9.7 vs 0.7) in the unselected sample, the selected sample included more winners at regional (39.9 vs. 12.0), federal (31.2 vs. 4.1), and international levels (6.3 vs. 2.7). Both measures of achievement were indeed linked with performance in Olympiads, with victory in Olympiad explaining 9 and 24 per cent of variance in Year grade and Examination, respectively. Significant differences were found between those who did not participate in Olympiads and those who won in any level Olympiad. No other differences were found.

We expected that winners at all Olympiad levels will receive excellent Year grades. However, we found that their average grade was below excellent. These findings also support the notion of the 5‐point teacher ratings being an imprecise measure of achievement, being too crude to tap into individual differences at the high end of achievement. In contrast, there were differences in Examination scores between city, regional, and federal level Olympiad winners, with no differences between those who won in school Olympiad and did not participate in Olympiad at all. Given that the complexity of tasks increases with each Olympiad level, the ability of Examination to differentiate between students winning at different levels supports the possibility that Examination is a more valid measure of achievement in comparison with Year grade. Our results also suggest that winning in Olympiad at a particular level (i.e., regional) can be used for identification of high ability and achievement potential, but need to be supplemented with other measures.

### Sex differences

The current study also examined sex differences in all investigated traits. As we found no interaction between factor of selection and sex, here we discuss sex differences irrespective to selected/unselected division. Females demonstrated higher agreeableness, conscientiousness, openness to experience, and neuroticism. These results are consistent with a number of previous studies showing that females report higher scores than males in agreeableness (Costa, Terracciano, & McCrae, 2001; Feingold, 1994; Rimfeld *et al.*, 2016; Weisberg *et al.*, 2011); conscientiousness (Costa *et al.*, 2001; Feingold, 1994; Rimfeld *et al.*, 2016); and neuroticism (Costa *et al.*, 2001; Rimfeld *et al.*, 2016; Weisberg *et al.*, 2011). However, some previous research did not find sex differences in openness to experience (Rimfeld *et al.*, 2016; Weisberg *et al.*, 2011), whereas this was detected in the current sample. In addition, females in our study did not differ from males in extraversion, which is in line with several previous studies (Costa *et al*, 2001; Feingold, 1994; Rimfeld *et al.*, 2016). Overall, the differences were small (ES* = *.01 to .09), with biggest effect for neuroticism (ES* = *.09). In line with previous findings, males in our study on average scored higher on psychopathy and Machiavellianism (Jonason & Davis, 2018; Matta *et al.*, 2019); and no sex differences emerged in narcissism (Jonason & Davis, 2018; Matta *et al.*, 2019).

In our data, females showed more prosocial behaviour (ES* = *.04), which is consistent with previous studies (e.g., Eren *et al.*, 2018). Females also scored higher on internalizing problems and total behavioural difficulties (ES* = *.04 and .03, respectively). These results are in line with several previous studies, which also found more behaviour difficulties in female adolescents (Eren *et al.*, 2018). In contrast, some studies reported more problems in males: externalizing problems (Eren *et al.*, 2018; Kristoffersen *et al.*, 2015) and overall behavioural difficulties (Morawska & Sanders, 2008). Differences in the results might have been driven by measures used: for example, Morawska and Sanders (2008) using parent‐report rather than child‐report measures; and sample characteristics: for example, generally younger participants (10–13 years old) in Kristoffersen *et al.* (2015) in comparison with the current study (14–17).

For achievement, females scored slightly higher in Year grade (ES* = *.04), but no differences were found in Year 9 State Examination. This again indirectly supports the hypothesis that personality characteristics may feed into the achievement grades. Females in our study were on average more prosocial and open to experience than males. People who are open to experience are often characterized as being curious, creative, and novelty seeking (Friedman & Schustack, 2009), which might be considered by teachers as preferable for education. In the current study, personality correlated with teacher grades but not with examination performance. Therefore, sex difference in teacher grades may stem from sex differences in personality that are not necessarily related to the actual achievement level. Of course, it is possible that the grades reflected true effects of personality (e.g., openness) on achievement, although it is not clear why this would not be true of examination performance. Future research is needed to clarify this pattern of results. It is also possible that the pattern of results would differ for different academic subjects. Previous studies (Freeman & Garces‐Bascal, 2015; Stoet & Geary, 2013) reported that males on average outperformed females in STEM subjects and non‐verbal assessments, whereas females on average outperformed males in verbal tests. We could not explore this in the current study due to the reduced variance in both Year grade and Year 9 State Examination (scale from 2 to 5). To compensate for the reduced variance, we combined scores for Maths and Russian, investigating sex differences in overall achievement. Future research should address this limitation.

### Conclusions and future directions

The present study overcomes some limitations of much previous research. The study was well powered and offered a direct comparison of selected and unselected groups. Overall, our data suggest absence of a particular trait that would characterize the gifted sample robustly, apart from achievement. These results provide further support for the view on giftedness as a complex phenomenon that includes an interacting networks of cognitive abilities and personality characteristics (see, e.g., Sternberg & Davidson, 2005). It is likely that there is no simple personality profile that leads to high performance, but rather a cumulative effect of multiple traits. This notion is probable especially in the light of recent data from behavioural genetics that showed: complex aetiology for all traits that includes interactions between genes and environments (Plomin *et al.*, 2016); multiple polymorphisms affecting all complex behaviours, for example, intelligence (Docherty, Kovas, Petrill & Plomin, 2010); and individual profile of cognitive and non‐cognitive traits for each person (Kovas, Malykh & Gaysina, 2016).

Differences in personality and behaviour between the samples might be in fact larger but were probably masked by the characteristics of unselected sample in the current study. Despite the relatively large sample size, representing unselected schools, it may still not be representative of the general population. For example, the sample contained high proportion of Olympiad winners, which may be indicative of higher than average level of intelligence. On the other hand, this may not be the case as this disproportionate Olympiad success was at the lower level (school‐ and city‐level). We cannot elaborate this hypothesis further as the study did not include any intelligence measures. However, we are confident that our STEM‐selected participants represent a truly high cognitive ability group. For example, one recent study (E. Tsigeman, M. Likhanov, A. Budakova, & Y. Kovas, unpublished data) demonstrated large advantage in spatial ability of another STEM‐selected sample from educational centre Sirius over the current unselected sample. In the current study, selection criteria for STEM sample were the same and the spatial ability data available for a small number of participants (*N = *56) showed similarly high performance.

It is also possible that stronger average differences in personality and psychological difficulties between gifted and unselected adolescents are present in particular domains of giftedness. Previous research found that openness to experience was more strongly associated with verbal rather than non‐verbal intelligence (as per Ashton, Lee, Vernon & Jang, 2000). In our study, selected adolescents were STEM‐focused. In addition, other personality traits, for example, motivation or values (Bernstain, Lubinski & Benbow, 2019), may be stronger predictors of higher achievement.

One source of inconsistencies across different studies might be differences in criteria for identification of selected (gifted) samples. In the current study, students were selected for admission to Sirius on their academic record; other studies used state guidelines for talent identification (Zeidner & Shani‐Zinovich, 2011), Wechsler Intelligence Scale for Children (WISC‐R, Eren *et al.*, 2018), or membership in specific associations (e.g., Gifted and Talented Associations; Morawska & Sanders, 2008). The criteria for unselected samples also differ. For example, Morawska and Sanders (2008) used population normative data as a reference sample; other studies collected data in several general education schools, for example, Zeidner and Shani‐Zinovich (2011), as was done in the present study. Longitudinal studies are needed to explore how the tiny group differences may grow over the course of students’ education and how they can relate to students’ future success. More collaborative research is needed, using the same measures and selection criteria and testing specific hypotheses regarding differences between gifted adolescents and their peers.

## Funding

This research received no external funding.

## Conflicts of interest

All authors declare no conflict of interest.

## Author contributions

Y.K., K.P., M.L., and E.T. conceptualized the study. K.P. contributed to methodology. E.T. and M.L. performed formal analysis. Y.K. and M.L. provided resources. M.L., A.A., and I.S. curated the data. M.L. and E.T. wrote the original draft of the manuscript. Y.K. and K.P. supervised the study. M.L. involved in project administration.

## Ethical approval

Code of ethical approval: 16012018‐5.

## Supporting information


**Table S1**. Correlations among study variables in selected sample.
**Table S2**. Correlations among study variables in unselected sample.

## Data Availability

The data that support the findings of this study are available from the corresponding author upon reasonable request.
