# Supplementary material for: Ordinary extraordinary: Elusive group differences in personality and psychological difficulties between STEM‐gifted adolescents and their peers
Source: Br J Educ Psychol. 2020 Apr 28;91(1):78–100. doi: 10.1111/bjep.12349 (PMC7983905; doi:10.1111/bjep.12349)
Supplement: Supplementary file 1 — Table S1. Correlations among study variables in selected sample. Table S2. Correlations among study variables in unselected sample. [file BJEP-91-78-s001.docx]

Appendix

| Table S1.Correlations among study variables in selected sample | | | | | | | | | | | | | |
| --- | --- | --- | --- | --- | --- | --- | --- | --- | --- | --- | --- | --- | --- |
|  |  |  |  |  |  |  |  |  |  |  |  |  | 13. |
| 1. Conscientiousness | – |  |  |  |  |  |  |  |  |  |  |  |  |
| 1. Extraversion | .310** | – |  |  |  |  |  |  |  |  |  |  |  |
| 1. Agreeableness | .308** | .267** | – |  |  |  |  |  |  |  |  |  |  |
| 1. Neuroticism | -.350** | -.429** | -.261** | – |  |  |  |  |  |  |  |  |  |
| 1. Openness to experience | .258** | .362** | .194** | -.068* | – |  |  |  |  |  |  |  |  |
| 1. Narcissism | .342** | .546** | .011 | -.236** | .384** | – |  |  |  |  |  |  |  |
| 1. Psychopathy | -.249** | .037 | -.524** | .168** | -.024 | .189** | – |  |  |  |  |  |  |
| 1. Machiavellianism | -.021 | -.087** | -.412** | .067* | -.007 | .200** | .405** | – |  |  |  |  |  |
| 1. Prosocial scale | .318** | .334** | .553** | -.072* | .319** | .135** | -.282** | -.286** | – |  |  |  |  |
| 1. Internalisingscale | -.284** | -.552** | -.256** | .634** | -.152** | -.301** | .134** | .128** | -.146** | – |  |  |  |
| 1. Externalising scale | -.569** | -.048 | -.346** | .413** | -.061 | -.066* | .432** | .101** | -.163** | .337** | – |  |  |
| 1. Total behavioural difficulties | -.508** | -.407** | -.366** | .656** | -.127** | -.247** | .339** | .158** | -.185** | .842** | .772** | – |  |
| 1. Year grade | .213** | .026 | .093** | .041 | .061 | .064 | -.132** | -.047 | .055 | .038 | -.124** | -.051 | – |
| *Note: **p<.01. *p<.05* | | | | | | | | | | | | | |

| Table S2.Correlations among study variables in unselected sample | | | | | | | | | | | | | |
| --- | --- | --- | --- | --- | --- | --- | --- | --- | --- | --- | --- | --- | --- |
|  | 1. | 2. | 3. | 4. | 5. | 6. | 7. | 8. | 9. | 10. | 11. | 12. | 13. |
| 1. Conscientiousness | – |  |  |  |  |  |  |  |  |  |  |  |  |
| 1. Extraversion | .469** | – |  |  |  |  |  |  |  |  |  |  |  |
| 1. Agreeableness | .379** | .312** | – |  |  |  |  |  |  |  |  |  |  |
| 1. Neuroticism | -.410** | -.444** | -.319** | – |  |  |  |  |  |  |  |  |  |
| 1. Openness to experience | .287** | .372** | .282** | -.120** | – |  |  |  |  |  |  |  |  |
| 1. Narcissism | .263** | .554** | .045 | -.206** | .414** | – |  |  |  |  |  |  |  |
| 1. Psychopathy | -.326** | -.060 | -.559** | .219** | -.113** | .156** | – |  |  |  |  |  |  |
| 1. Machiavellianism | -.054 | -.029 | -.321** | .083* | .052 | .240** | .391** | – |  |  |  |  |  |
| 1. Prosocial scale | .365** | .342** | .565** | -.173** | .303** | .163** | -.353** | -.226** | – |  |  |  |  |
| 1. Internalisingscale | -.422** | -.574** | -.297** | .594** | -.153** | -.304** | .162** | .139** | -.230** | – |  |  |  |
| 1. Externalising scale | -.540** | -.107** | -.418** | .424** | -.149** | .036 | .493** | .130** | -.303** | .385** | – |  |  |
| 1. Total behavioural difficulties | -.575** | -.443** | -.418** | .629** | -.178** | -.185** | .383** | .167** | -.312** | .861** | .788** | – |  |
| 1. Year grade | .206** | .037 | .113** | .031 | .132** | .064 | -.157** | -.004 | .096** | -.070* | -.174** | -.142** | – |
| *Note: **p<.01. *p<.05* | | | | | | | | | | | | | |
